# “Shadow of a Doubt”—The Pathogenic Role of Endometrial Defects in Endometriosis Development and Endometriosis-Associated Infertility: Robust Demonstration of Clinical Relevance Is Still Urgently Needed †

**DOI:** 10.3390/biom13040651

**Published:** 2023-04-05

**Authors:** Paola Viganò, Maíra Casalechi, Paolo Vercellini, Edgardo Somigliana

**Affiliations:** 1 Infertility Unit, Fondazione IRCCS Ca’ Granda Ospedale Maggiore Policlinico, Via F. Sforza 28, 20122 Milan, Italy; 2 Division of Human Reproduction, Hospital das Clínicas-Universidade Federal de Minas Gerais, Belo Horizonte 30130-100, Brazil; mairacasalechi@gmail.com; 3 Department of Clinical Sciences and Community Health, Università degli Studi di Milano, 20122 Milan, Italy; paolo.vercellini@unimi.it (P.V.); edgardo.somigliana@policlinico.mi.it (E.S.); 4 Gynecology Unit, Fondazione IRCCS Ca’ Granda Ospedale Maggiore Policlinico, 20122 Milan, Italy

Endometriosis is an estrogen-dependent chronic inflammatory disease characterized by the presence of endometrial glands and stroma associated with fibrosis outside the uterine cavity [1]. Painful symptoms and/or infertility are typically associated with the disease.

Hundreds of molecules have been reported to be differentially expressed at gene and/or protein levels in the eutopic endometrium of patients with endometriosis compared to that of non-affected women [2,3,4,5,6,7,8]. These aberrations have been observed in all the steps involved in the cellular processes from menstrual shedding to the occurrence of endometriosis. Endometrial defects have been demonstrated for molecules and genes involved in proteolysis, as well as ability to evade immunosurveillance, apoptosis, adhesion, proliferation, invasive potential, estrogen production, steroid hormone response, and angiogenesis. Overall, they have been used to explain the reasons underlying the 10% prevalence of the disease notwithstanding retrograde menstruation, at the basis of the most accepted pathogenetic theory, is a nearly universal phenomenon [1]. In other words, the fact that the eutopic endometrium of affected women shows alterations that are not found in the endometrium of disease-free women has prompted the view that the primary defects underlying the origin of the disease, would be in the eutopic endometrium itself or in the uterus [2]. Cells and tissue elements derived from such an altered eutopic endometrium would be more able to develop into endometriosis.

With this idea, the general thinking is that the endometrium of women with endometriosis is also less receptive to embryo implantation [9]. Endometrial changes typical of affected patients, primarily or secondarily to the inflammation associated with the disease, may be responsible, at least partially, for defective receptivity. Indeed, endometrial aberrations have been demonstrated for molecules and genes involved in embryo adhesion and invasion, decidualization, pregnancy-induced immunologic changes, receptivity-associated cytokine milieu, steroid hormone responses, and angiogenesis during embryo implantation [2,3,4,5,6,7,8].

In the paper entitled ‘From retrograde menstruation to endometrial determinism and a brave new world of “root treatment” of endometriosis: the destiny or a fanciful utopia?’, Guo and coworkers have provided a critical appraisal of the evidence supporting endometrial aberrations in women with endometriosis with a particular focus on their clinical relevance [10]. The authors have emphasized the idea that many of these alterations are not present in all women with endometriosis, nor are they exclusively present in affected women. They also underlined that the available evidence supporting endometrial changes in women with endometriosis came from patients who have already been diagnosed with the disease. Therefore, eutopic endometrium may acquire these aberrations secondarily, as a sort of epiphenomenon of disease development. Furthermore, a phylogenetic relation between endometrial aberrations and the disease has not been unequivocally demonstrated. More importantly, in order to establish that endometriosis originates from the alteration-carrying endometrium, one would have to demonstrate that endometrial tissues carrying these alterations confer a significantly higher risk of developing the disease compared to those that do not. However, this has not yet been proven. Similarly, for the defective receptivity, one would have to demonstrate that endometrial tissues carrying such alterations confer a significantly higher risk of reduced embryo implantation compared to those that do not. Studies addressing infertility status in women with endometriosis after rigorously controlling for ovarian and embryo parameters do not support this view. When comparing women with endometriosis, non-infertile patients who underwent assisted reproduction to test embryos for a single-gene disorder, and couples with isolated male factor infertility after euploid frozen embryo transfers, Bishop et al. found no differences in clinical pregnancy, pregnancy loss, or live birth rates among the three groups [11]. More interestingly, in most studies on recipients of oocyte donation affected by endometriosis entering the program due to low responses to gonadotropins or repeated IVF failure, cycle outcome parameters were reported to be similar to those of women with other reproductive disorders, raising questions on whether the uterine environment of women with endometriosis may affect the implantation process [12].

Overall, in the last 15 years, the endometrium of women with endometriosis has been the focus of intense research. Despite these efforts, robust explanations of the mechanisms underlying endometriosis development and associated infertility are still lacking. This is partly because we are still uncertain about the biological or clinical relevance of the endometrial changes observed in these women. As mentioned by Guo and co-workers, “*this may change soon*” [10]. In the meantime, the models used so far to assess the functional role of these aberrations in the context of disease establishment should be considered with a healthy degree of skepticism. At present, speculative arguments on ‘endometrial determinism’ should still be slightly moderated when presenting this kind of data.
